# (1*Z*)-1-(2,4-Dichloro­phen­yl)ethan-1-one semicarbazone

**DOI:** 10.1107/S1600536809029900

**Published:** 2009-08-08

**Authors:** Hoong-Kun Fun, Kasthuri Balasubramani, A. M. Vijesh, Shridhar Malladii, Arun M. Isloor

**Affiliations:** a X-ray Crystallography Unit, School of Physics, Universiti Sains Malaysia, 11800 Universiti Sains Malaysia, Penang, Malaysia; b SeQuent Scientific Limited, No. 120 A&B, Industrial Area, Baikampady, New Bangalore, Karnataka 575 011, India; c Department of Chemistry, National Institute of Technology-Karnataka, Surathkal, Mangalore 575 025, India

## Abstract

In the title compound, C_9_H_9_Cl_2_N_3_O, the semicarbazone group is approximately planar, with an r.m.s deviation from the mean plane of 0.011 (2) Å. The dihedral angle between the least-squares planes through the semicarbazone group and the benzene ring is 38.76 (9)°. The crystal structure is further stabilized by N—H⋯O and C—H⋯O hydrogen bonding.

## Related literature

For applications of semicarbazone derivatives, see: Warren *et al.* (1977 ▶); Chandra & Gupta (2005 ▶); Jain *et al.* (2002 ▶); Pilgram (1978 ▶); Yogeeswari *et al.* (2004 ▶); For semicarbazide preparations, see: Furniss *et al.* (1978 ▶). For hydrogen-bond motifs, see: Bernstein *et al.* (1995 ▶).
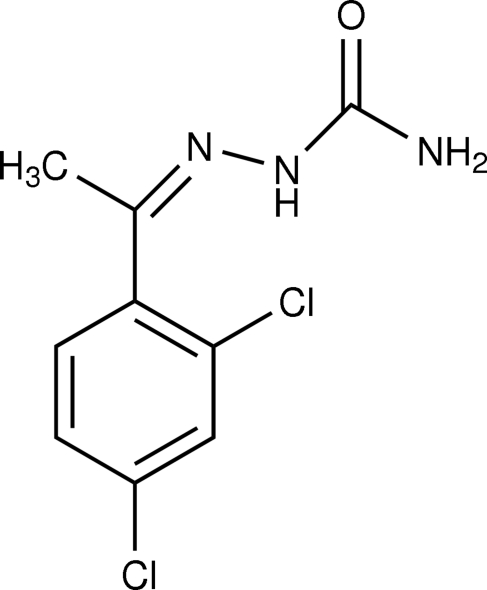


## Experimental

### 

#### Crystal data

C_9_H_9_Cl_2_N_3_O
*M* *_r_* = 246.09
Monoclinic, 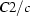

*a* = 37.8079 (17) Å
*b* = 3.8097 (2) Å
*c* = 14.4920 (7) Å
β = 98.852 (2)°
*V* = 2062.52 (17) Å^3^
*Z* = 8
Mo *K*α radiation
μ = 0.60 mm^−1^
*T* = 100 K
0.42 × 0.14 × 0.04 mm

#### Data collection

Bruker SMART APEXII CCD area-detector diffractometer
Absorption correction: multi-scan (*SADABS*; Bruker, 2005 ▶) *T*_min_ = 0.707, *T*_max_ = 0.974
32124 measured reflections
4202 independent reflections
3654 reflections with *I* > 2σ(*I*)
*R*_int_ = 0.037

#### Refinement

*R*[*F* ^2^ > 2σ(*F* ^2^)] = 0.072
*wR*(*F* ^2^) = 0.192
*S* = 1.11
4202 reflections
149 parameters
H atoms treated by a mixture of independent and constrained refinement
Δρ_max_ = 3.37 e Å^−3^
Δρ_min_ = −0.82 e Å^−3^

### 

Data collection: *APEX2* (Bruker, 2005 ▶); cell refinement: *SAINT* (Bruker, 2005 ▶); data reduction: *SAINT*; program(s) used to solve structure: *SHELXTL* (Sheldrick, 2008 ▶); program(s) used to refine structure: *SHELXTL*; molecular graphics: *SHELXTL*; software used to prepare material for publication: *SHELXTL* and *PLATON* (Spek, 2009 ▶).

## Supplementary Material

Crystal structure: contains datablocks global, I. DOI: 10.1107/S1600536809029900/bq2153sup1.cif

Structure factors: contains datablocks I. DOI: 10.1107/S1600536809029900/bq2153Isup2.hkl

Additional supplementary materials:  crystallographic information; 3D view; checkCIF report

## Figures and Tables

**Table 1:** Hydrogen-bond geometry (Å, °)

*D*—H⋯*A*	*D*—H	H⋯*A*	*D*⋯*A*	*D*—H⋯*A*
N2—H1*N*2⋯O1^i^	0.85 (3)	2.07 (3)	2.907 (2)	168 (3)
N3—H2*N*3⋯O1^ii^	0.81 (4)	2.13 (4)	2.924 (2)	164 (4)
C9—H9*A*⋯O1^iii^	0.96	2.59	3.465 (2)	152

Symmetry codes: (i) 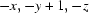; (ii) 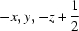; (iii) 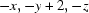.

**Fig. 1.**
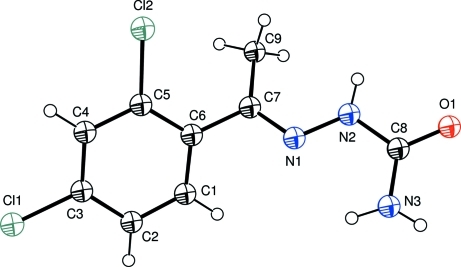
The molecular structure of (I), showing 50% probability displacement ellipsoids and the atom numbering scheme.

**Fig. 2.**
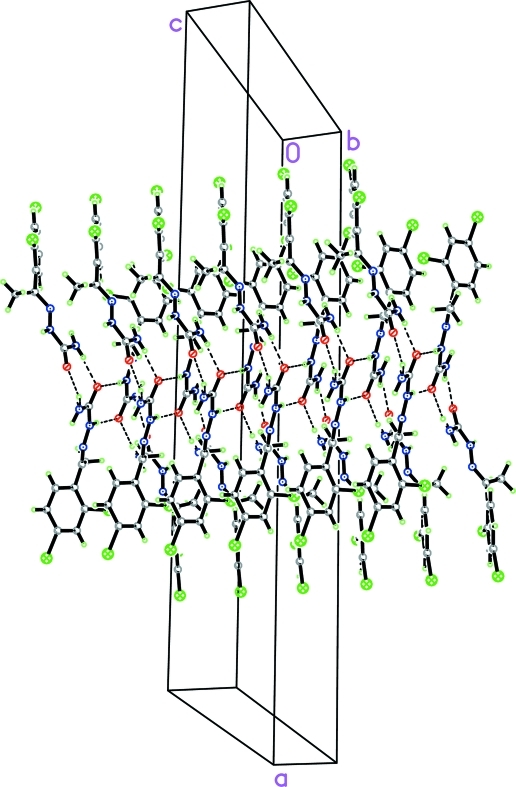
An one-dimensional chain of (I) with R22(8) ring motifs along the [010] direction. Dashed lines indicate the hydrogen bonding.

## supplementary crystallographic information

### Comment

In organic chemistry, a semicarbazone is a derivative of an aldehyde or ketone formed by a condensation between a ketone or aldehyde and semicarbazide. They find immense applications in the field of synthetic chemistry, such as medicinal chemistry (Warren *et al.*, 1977), organometallics (Chandra & Gupta, 2005), polymers (Jain *et al.*, 2002) and herbicides (Pilgram, 1978). 4-Sulphamoylphenyl semicarbazones were synthesized and were found to possess anticonvulsant activity (Yogeeswari *et al.*, 2004). Keeping in view of their biological importance, we hereby reporting crystal structure of the semicarbazone of commercial importance.

The semicarbazone group (Fig. 1) (C9/C6/C7/N1/N2/C8/O1/N3) is approximately planar, with an r.m.s deviation of 0.011 (2)Å for atom N1, while the dihedral angle between the least-squares plane through the semicarbazone group and the benzene ring is 38.76 (9)°. The molecules are linked *via* N—H···O hydrogen bonds to generate *R*_2_^2^(8) ring motifs (Bernstein *et al.* 1995). These motifs are further connected through C—H···O hydrogen bonds to form a one-dimensional chain along the [0 1 0] direction (Fig. 2).

### Experimental

3.16 g (28.3 mmol) of semicarbazide hydrochloride and 2.83 g (34.5 mmol) of crystallized sodium acetate was dissolved in 25 ml of water (Furniss *et al.*, 1978). The reaction mixture was stirred at room temperature for 10 minutes. (5.0 g, 26.5 mmol) of 2,4-dichloroacetophenone in 25 ml of ethanol was then added and the mixture stirred well for 6 h. The separated semicarbazone was filtered, washed with chilled water and recrystallized from an ethanol-DMF mixture. Yield was found to be 5.23 g, 86.02%. *M.p.* 501–503 K.

### Refinement

H atoms were positioned geometrically (C—H = 0.93–0.96 Å) and refined using a riding model with *U*_iso_(H) = 1.2*U*_eq_(C) and 1.5*U*_eq_(methyl C). A rotating–group model was used for the methyl groups. The nitrogen H atoms were located from the difference Fourier map [N–H = 0.85 (3)–0.91 (3) Å] and allowed to refine freely.

### Crystal data

**Table:**

C_9_H_9_Cl_2_N_3_O	*F*(000) = 1008
*M**_r_* = 246.09	*D*_x_ = 1.585 Mg m^−^^3^
Monoclinic, *C*2/*c*	Mo *K*α radiation, λ = 0.71073 Å
Hall symbol: -C 2yc	Cell parameters from 9961 reflections
*a* = 37.8079 (17) Å	θ = 2.9–34.0°
*b* = 3.8097 (2) Å	µ = 0.60 mm^−^^1^
*c* = 14.4920 (7) Å	*T* = 100 K
β = 98.852 (2)°	Plate, colorless
*V* = 2062.52 (17) Å^3^	0.42 × 0.14 × 0.04 mm
*Z* = 8	

### Data collection

**Table:**

Bruker SMART APEXII CCD area-detector diffractometer	4202 independent reflections
Radiation source: fine-focus sealed tube	3654 reflections with *I* > 2σ(*I*)
graphite	*R*_int_ = 0.037
φ and ω scans	θ_max_ = 34.1°, θ_min_ = 1.1°
Absorption correction: multi-scan (*SADABS*; Bruker, 2005)	*h* = −59→58
*T*_min_ = 0.707, *T*_max_ = 0.974	*k* = −5→5
32124 measured reflections	*l* = −22→22

### Refinement

**Table:**

Refinement on *F*^2^	Primary atom site location: structure-invariant direct methods
Least-squares matrix: full	Secondary atom site location: difference Fourier map
*R*[*F*^2^ > 2σ(*F*^2^)] = 0.072	Hydrogen site location: inferred from neighbouring sites
*wR*(*F*^2^) = 0.192	H atoms treated by a mixture of independent and constrained refinement
*S* = 1.11	*w* = 1/[σ^2^(*F*_o_^2^) + (0.1176*P*)^2^ + 5.115*P*] where *P* = (*F*_o_^2^ + 2*F*_c_^2^)/3
4202 reflections	(Δ/σ)_max_ = 0.001
149 parameters	Δρ_max_ = 3.37 e Å^−^^3^
0 restraints	Δρ_min_ = −0.82 e Å^−^^3^

### Special details

**Table:**

Experimental. The crystal was placed in the cold stream of an Oxford Cryosystems Cobra open-flow nitrogen cryostat (Cosier & Glazer, 1986) operating at 100.0 (1) K.
Geometry. All s.u.'s (except the s.u. in the dihedral angle between two l.s. planes) are estimated using the full covariance matrix. The cell s.u.'s are taken into account individually in the estimation of s.u.'s in distances, angles and torsion angles; correlations between s.u.'s in cell parameters are only used when they are defined by crystal symmetry. An approximate (isotropic) treatment of cell s.u.'s is used for estimating s.u.'s involving l.s. planes.
Refinement. Refinement of F^2^ against ALL reflections. The weighted R-factor wR and goodness of fit S are based on F^2^, conventional R-factors R are based on F, with F set to zero for negative F^2^. The threshold expression of F^2^ > σ(F^2^) is used only for calculating R-factors(gt) etc. and is not relevant to the choice of reflections for refinement. R-factors based on F^2^ are statistically about twice as large as those based on F, and R- factors based on ALL data will be even larger.

### Fractional atomic coordinates and isotropic or equivalent isotropic displacement parameters (Å^2^)

**Table:**

	*x*	*y*	*z*	*U*_iso_*/*U*_eq_	
Cl1	0.254197 (12)	1.14825 (12)	0.14159 (3)	0.01486 (13)	
Cl2	0.149699 (12)	0.64254 (12)	−0.11406 (3)	0.01413 (13)	
O1	−0.00572 (4)	0.4635 (5)	0.11828 (10)	0.0162 (3)	
N1	0.08094 (4)	0.7191 (5)	0.08948 (11)	0.0127 (3)	
N2	0.04458 (4)	0.6666 (5)	0.06771 (12)	0.0141 (3)	
N3	0.04484 (5)	0.5433 (7)	0.22395 (13)	0.0222 (4)	
C1	0.14889 (5)	1.0482 (6)	0.14140 (13)	0.0132 (3)	
H1A	0.1327	1.0954	0.1819	0.016*	
C2	0.18500 (5)	1.1187 (5)	0.17051 (14)	0.0132 (3)	
H2A	0.1928	1.2131	0.2293	0.016*	
C3	0.20913 (5)	1.0451 (5)	0.10974 (13)	0.0120 (3)	
C4	0.19805 (5)	0.9013 (5)	0.02261 (13)	0.0124 (3)	
H4A	0.2145	0.8508	−0.0172	0.015*	
C5	0.16165 (5)	0.8336 (5)	−0.00436 (13)	0.0112 (3)	
C6	0.13617 (5)	0.9086 (5)	0.05322 (13)	0.0112 (3)	
C7	0.09697 (5)	0.8539 (5)	0.02541 (13)	0.0116 (3)	
C8	0.02647 (5)	0.5559 (6)	0.13727 (14)	0.0152 (3)	
C9	0.07833 (5)	0.9765 (5)	−0.06811 (13)	0.0123 (3)	
H9A	0.0571	1.1034	−0.0601	0.018*	
H9B	0.0720	0.7772	−0.1078	0.018*	
H9C	0.0940	1.1272	−0.0962	0.018*	
H1N2	0.0320 (9)	0.660 (8)	0.014 (2)	0.016 (7)*	
H1N3	0.0663 (10)	0.636 (10)	0.231 (3)	0.028 (9)*	
H2N3	0.0324 (10)	0.484 (10)	0.262 (3)	0.028 (9)*	

### Atomic displacement parameters (Å^2^)

**Table:**

	*U*^11^	*U*^22^	*U*^33^	*U*^12^	*U*^13^	*U*^23^
Cl1	0.0092 (2)	0.0180 (2)	0.0168 (2)	−0.00196 (13)	0.00036 (15)	−0.00011 (14)
Cl2	0.0141 (2)	0.0179 (2)	0.0105 (2)	−0.00169 (14)	0.00232 (15)	−0.00251 (14)
O1	0.0090 (6)	0.0283 (8)	0.0111 (6)	−0.0027 (5)	0.0010 (5)	0.0002 (5)
N1	0.0076 (6)	0.0192 (7)	0.0111 (7)	−0.0008 (5)	0.0009 (5)	0.0005 (5)
N2	0.0084 (6)	0.0238 (8)	0.0097 (7)	−0.0026 (5)	0.0005 (5)	0.0012 (5)
N3	0.0114 (7)	0.0461 (12)	0.0088 (7)	−0.0060 (8)	0.0002 (6)	0.0043 (7)
C1	0.0100 (7)	0.0191 (8)	0.0104 (7)	−0.0019 (6)	0.0016 (6)	−0.0020 (6)
C2	0.0114 (7)	0.0163 (8)	0.0114 (7)	−0.0007 (6)	0.0005 (6)	−0.0018 (6)
C3	0.0082 (7)	0.0140 (8)	0.0135 (8)	−0.0007 (6)	0.0006 (6)	0.0011 (6)
C4	0.0119 (7)	0.0126 (7)	0.0130 (8)	−0.0005 (6)	0.0027 (6)	−0.0004 (6)
C5	0.0113 (7)	0.0127 (7)	0.0097 (7)	0.0001 (5)	0.0021 (6)	−0.0005 (5)
C6	0.0104 (7)	0.0130 (7)	0.0101 (7)	−0.0007 (6)	0.0015 (5)	0.0013 (6)
C7	0.0099 (7)	0.0141 (8)	0.0105 (7)	−0.0007 (5)	0.0009 (6)	−0.0003 (5)
C8	0.0109 (7)	0.0234 (9)	0.0116 (8)	−0.0011 (7)	0.0025 (6)	0.0017 (7)
C9	0.0107 (7)	0.0140 (8)	0.0114 (7)	−0.0011 (6)	−0.0010 (6)	0.0017 (6)

### Geometric parameters (Å, °)

**Table:**

Cl1—C3	1.7403 (19)	C1—H1A	0.9300
Cl2—C5	1.7436 (19)	C2—C3	1.391 (3)
O1—C8	1.256 (2)	C2—H2A	0.9300
N1—C7	1.291 (2)	C3—C4	1.381 (3)
N1—N2	1.377 (2)	C4—C5	1.395 (3)
N2—C8	1.369 (2)	C4—H4A	0.9300
N2—H1N2	0.85 (3)	C5—C6	1.398 (3)
N3—C8	1.339 (3)	C6—C7	1.489 (3)
N3—H1N3	0.88 (4)	C7—C9	1.503 (3)
N3—H2N3	0.81 (4)	C9—H9A	0.9600
C1—C2	1.392 (3)	C9—H9B	0.9600
C1—C6	1.399 (3)	C9—H9C	0.9600
			
C7—N1—N2	117.12 (16)	C4—C5—C6	122.38 (17)
C8—N2—N1	118.08 (16)	C4—C5—Cl2	116.02 (14)
C8—N2—H1N2	113 (2)	C6—C5—Cl2	121.59 (15)
N1—N2—H1N2	128 (2)	C5—C6—C1	116.83 (17)
C8—N3—H1N3	115 (2)	C5—C6—C7	123.95 (17)
C8—N3—H2N3	112 (3)	C1—C6—C7	119.21 (16)
H1N3—N3—H2N3	131 (4)	N1—C7—C6	114.76 (16)
C2—C1—C6	122.24 (17)	N1—C7—C9	124.43 (17)
C2—C1—H1A	118.9	C6—C7—C9	120.65 (16)
C6—C1—H1A	118.9	O1—C8—N3	122.71 (18)
C3—C2—C1	118.53 (17)	O1—C8—N2	120.16 (18)
C3—C2—H2A	120.7	N3—C8—N2	117.11 (18)
C1—C2—H2A	120.7	C7—C9—H9A	109.5
C4—C3—C2	121.52 (17)	C7—C9—H9B	109.5
C4—C3—Cl1	118.62 (14)	H9A—C9—H9B	109.5
C2—C3—Cl1	119.83 (15)	C7—C9—H9C	109.5
C3—C4—C5	118.48 (17)	H9A—C9—H9C	109.5
C3—C4—H4A	120.8	H9B—C9—H9C	109.5
C5—C4—H4A	120.8		
			
C7—N1—N2—C8	−173.82 (19)	Cl2—C5—C6—C7	−3.6 (3)
C6—C1—C2—C3	−0.4 (3)	C2—C1—C6—C5	1.6 (3)
C1—C2—C3—C4	−0.7 (3)	C2—C1—C6—C7	−177.46 (18)
C1—C2—C3—Cl1	177.08 (15)	N2—N1—C7—C6	179.31 (17)
C2—C3—C4—C5	0.7 (3)	N2—N1—C7—C9	4.0 (3)
Cl1—C3—C4—C5	−177.15 (14)	C5—C6—C7—N1	137.72 (19)
C3—C4—C5—C6	0.5 (3)	C1—C6—C7—N1	−43.3 (3)
C3—C4—C5—Cl2	−178.54 (15)	C5—C6—C7—C9	−46.8 (3)
C4—C5—C6—C1	−1.6 (3)	C1—C6—C7—C9	132.2 (2)
Cl2—C5—C6—C1	177.39 (15)	N1—N2—C8—O1	−171.2 (2)
C4—C5—C6—C7	177.37 (18)	N1—N2—C8—N3	7.1 (3)

### Hydrogen-bond geometry (Å, °)

**Table:**

*D*—H···*A*	*D*—H	H···*A*	*D*···*A*	*D*—H···*A*
N2—H1N2···O1^i^	0.85 (3)	2.07 (3)	2.907 (2)	168 (3)
N3—H2N3···O1^ii^	0.81 (4)	2.13 (4)	2.924 (2)	164 (4)
C9—H9A···O1^iii^	0.96	2.59	3.465 (2)	152

Symmetry codes: (i) −*x*, −*y*+1, −*z*; (ii) −*x*, *y*, −*z*+1/2; (iii) −*x*, −*y*+2, −*z*.
